# Glucose-6-phosphate dehydrogenase deficiency among malaria suspects attending Gambella hospital, southwest Ethiopia

**DOI:** 10.1186/1475-2875-13-438

**Published:** 2014-11-18

**Authors:** Arega Tsegaye, Lemu Golassa, Hassen Mamo, Berhanu Erko

**Affiliations:** Department of Microbial, Cellular and Molecular Biology, College of Natural Sciences, Addis Ababa University, P. O. Box 1176, Addis Ababa, Ethiopia; Jimma University, P. O. Box 378, Jimma, Ethiopia; Aklilu Lemma Institute of Pathobiology, Addis Ababa University, P.O. Box 1176, Addis Ababa, Ethiopia; Armauer Hansen Research Institute, P. O. Box 1005, Addis Ababa, Ethiopia

**Keywords:** Malaria, Glucose-6-phosphate dehydrogenase deficiency, Phenotype, Prevalence, Gambella, Ethiopia

## Abstract

**Background:**

Glucose-6-phosphate dehydrogenase deficiency (G6PDd) is widespread across malaria endemic regions. G6PD-deficient individuals are at risk of haemolysis when exposed, among other agents, to primaquine and tafenoquine, which are capable of blocking malaria transmission by killing *Plasmodium falciparum* gametocytes and preventing *Plasmodium vivax* relapses by targeting hypnozoites. It is evident that no measures are currently in place to ensure safe delivery of these drugs within the context of G6PDd risk. Thus, determining G6PDd prevalence in malarious areas would contribute towards avoiding possible complications in malaria elimination using the drugs. This study, therefore, was aimed at determining G6PDd prevalence in Gambella hospital, southwest Ethiopia, using CareStart™ G6PDd fluorescence spot test.

**Methods:**

Venous blood samples were collected from febrile patients (n = 449) attending Gambella hospital in November-December 2013. Malaria was diagnosed using blood films and G6PDd was screened using CareStart™ G6PDd screening test (Access Bio, New Jersey, USA). Haematological parameters were also measured. The association of G6PD phenotype with sex, ethnic group and malaria smear positivity was tested.

**Results:**

Malaria prevalence was 59.2% (96.6% of the cases being *P. falciparum* mono infections). Totally 33 participants (7.3%) were G6PD-deficient with no significant difference between the sexes. The chance of being G6PD-deficient was significantly higher for the native ethnic groups (*Anuak* and *Nuer*) compared to the ‘highlanders’/settlers (odds ratio (OD) = 3.9, 95% confidence interval (CI) 0.481-31.418 for *Anuak* vs ‘highlanders’; OD = 4.9, 95% CI 0.635-38.00 for *Nuer* vs ‘highlanders’). G6PDd prevalence among the *Nuer* (14.3%) was significantly higher than that for the *Anuak* (12.0%).

**Conclusions:**

G6PDd prevalence in the area is substantial with 30 (90.9%) of the 33 deficient individuals having malaria suggesting the non-protective role of the disorder at least from clinical malaria. The indigenous Nilotic people tend to have a higher chance of being G6PD-deficient as 32 (96.9%) of the total 33 cases occurred among them.

## Background

Despite some reduction in the global burden of malaria in recent years [1], the disease still stands a grave public health concern. Apart from multiple environmental and parasite factors that complicate human-*Plasmodium* interactions, human genetic background confers additional complexity in the fight against malaria [2]. A high degree of variation exists between human populations and individuals with respect to malaria incidence, parasite density and disease severity and reactions to anti-malarials [3]. Although in some studies glucose-6-phosphate dehydrogenase deficiency (G6PDd) is associated with protection from severe malaria [4, 5] deficient individuals are reported to suffer from haemolytic anaemia when treated with primaquine [6], which is the only available drug against *Plasmodium falciparum* gametocytes [7] and a radical cure of *Plasmodium vivax* [8]. Further individuals with G6DPd are reported to suffer from haemolysis when treated with tafenoquine, another anti-malarial with proposed indication of treating hypnozoites of *P. vivax* and *Plasmodium ovale* [9].

G6PDd is one of the most common human enzymopathies which is in a very high frequency in tropical and subtropical malaria endemic regions [10, 11]. In sub-Saharan Africa (SSA), the prevalence of G6PDd can reach up to 35% although organized reports are lacking and different allelic variants are unevenly distributed in the region [12]. However, with progressive improvements in detection methods past G6PDd prevalence figures may need continuous revision. Population screening is recommended in regions where G6PDd prevalence is ≥3–5% to decide on the use of primaquine/tafenoquine and minimize the risk of primaquine/tefenoquine-induced complications in malaria elimination schemes [13]. In Ethiopia, past population-based G6PDd studies are limited and the practice of G6PDd screening in healthcare settings is also missing. As a result, vital information on G6PDd prevalence is lacking in the country, especially in malaria endemic foci. This study, therefore, was aimed at bridging the gap by diagnosing G6PDd among malaria suspects attending Gambella hospital, southwest Ethiopia, using a qualitative rapid G6PDd screening kit.

## Methods

### Study area

The study was conducted in Gambella hospital, Gambella Town. The town is the administrative center of Gambella Regional State and is located in the southwest of Ethiopia at about 777 km from Addis Ababa. Gambella hospital which serves an estimated 200,000 patients is the only hospital in the Region when this study was conducted. Gambella Region borders with Benishangul Gumuz and Oromiya Regions in the north; with the Southern Nations, Nationalities and Peoples’ Region (SNNPR) and the Sudan to the south; Oromiya and SNNPR to the east and the Sudan to the west. The region has an area of 25,800 km^2^ with an estimated total population of 42,474 having six major native Nilotic ethnic groups, namely, *Anuak* (also called *Anywaa*), *Nuer*, *Majenger*, *Opo*, *Surma* and *Komo* [14]. The *Nuer* and the *Anuak* are the dominant groups in the region and in Gambella Town settlers of various ethnic origins who moved from all over Ethiopia are loosely defined as “highlanders”.

The town is a lowland area with average altitude of 300–500 metres above sea level and has latitude and longitude of 8°15′00″ N and 34°34′59″ E, respectively. The main rainy season of the region is between May and October, and from November through April it is mostly dry [15]. According to this same source [15], the mean annual temperature of the region ranges from 17.3 to 28.3°C and the annual monthly temperature vary between 27 and 33°C throughout the year. The absolute maximum and minimum temperatures are 45.0 and 10.3°C occurring in mid-March and December, respectively. The average yearly rainfall is 1200 millimetre. Subsistence cultivation of maize and sorghum along the sides of the major rivers - Baro, Akobo and Gilo - is practiced by the *Anuak*. Fishing and forest hunting may be supplementary income sources. Most *Nuers* who mainly live in the driest Ethio-Sudanese border of the region largely depend on livestock [16].

Gambella is characterized by high malaria transmission due to its suitable climate and high rainfall. *P. falciparum* and *P. vivax* contribute to most of the malaria cases of the general population and *Anopheles gambiae* and *Anopheles pharoensis* are the primary mosquito vector species [17] *.* Some *Plasmodium malariae* cases were also reported from Gambella in an extensive epidemiological study in the late 1960s [18].

### Study population and design

Asymptomatic individuals for G6PDd but suspected of malaria (axillary temperature ≥37.5°C) were recruited in a hospital-based cross-sectional study between mid-November and December 2013. Patients who had received anti-malarial treatment within the past 48 hours and those having chronic illnesses were excluded. The minimum sample size (n) for the study was calculated using the formula  [19]; where ‘Z’ is standard normal distribution curve value for the 95% confidence interval (CI) (Z = 1.96), ‘p’ estimate prevalence (0.5%) and ‘m’ 5% margin of error (0.05). With a response rate of 96%, the estimated sample size was 400.

### Sample collection and analysis

After a questionnaire and clinical examination, 5 ml venous blood was collected in ethylene diamine tetra acetic acid (EDTA) for measuring haematological parameters, malaria diagnosis and G6PDd screening. The haematological parameters - haemoglobin (Hb), haematocrit (HCT) and packed cell volume (PCV) - and red cell indices (mean corpuscular volume (MCV), mean corpuscular haemoglobin (MCH) and mean corpuscular haemoglobin concentration (MCHC) were determined using an automated system, CELL-DYN 1400 haematology analyzer (Abbott, USA). Thick and thin blood smears were prepared and stained using 10% Giemsa solution to detect malaria. The stained slides were examined under a light microscope using the oil immersion objective (100X). Two experienced laboratory technologists independently screened the slides and discrepancies were settled by a third person’s blind test. Parasite density was calculated per 200 white blood cells (WBC) assuming 8,000 WBC/μl of blood [20]. G6PDd was screened using CareStart™ G6PDd screening test (Access Bio, Inc., New Jersey, USA) following the manufacturer’s instruction. Briefly, a 2 μl whole blood was added to a sample well and then 100 μl of assay buffer to the buffer well immediately after application of blood. The result was read normal as distinct purple colour, deficient as no colour or a very faint purple colour; and invalid when there is no blood migration or incomplete blood migration in the reading window within 10 minutes.

### Data analysis

Data were entered in Microsoft Excel spreadsheet, checked for correctness and completeness, and exported to and analysed using statistical package for social sciences (SPSS) version 20 (SPSS IBM, Chicago, IL). The Chi-squared (*X*^2^) test was used to determine the association between occurrence of malaria and G6PD status. Difference between means of haematological parameters for G6PD-deficent and normal individuals was analysed by analysis of variance (ANOVA). The logistic regression model was used to determine the relationship between sex and ethnic group with G6PD-deficent phenotype.

### Ethical clearance

The study obtained ethical clearance from College of Natural Sciences Institutional Ethics Review Board, Addis Ababa University. The study participants were recruited after providing their voluntary informed consent. Health personnel examined the patients, collected blood sample and treated malaria positives free of charge as per the national treatment guideline.

## Results

Overall, 510 febrile patients were prescribed for malaria blood smear during the study period. Fourteen individuals who declined to participate and 47 who had received anti-malarial treatment within the past 48 hours prior to sample collection were excluded. Thus blood samples from 449 patients, 210 males and 239 females, were analysed. The mean age was 16 years, median 15 and range 1–90 years; and mean haemoglobin (Hb) was 14.02 g/dl and median 13.3 g/dl, with no significant difference between the sexes. Overall, 266(59.2%) individuals were malaria smear positive with 257(96.6%) *P. falciparum* mono, 7(2.6%) *P. vivax* mono and 2(0.75%) *P. falciparum/P. vivax* mixed infections. Thirty three (7.3%) individuals were deficient for G6PD enzyme activity (Table 1). Although the prevalence of G6PDd was slightly higher among males (8.6%) than females (6.3%), the difference was not statistically significant (*p* = 0.354). In a univariate logistic analysis, the odds of being G6PD-deficient were 1.4 times higher for males [95% CI 0.678-2.853] than for females but the difference was not statistically significant (*p* > 0.05). However, the occurrence of G6PDd varied between ethnic groups. A greater proportion of the *Nuer* were G6PD-deficient (14.3%) compared to the ‘highlanders’ combined, who were all non-deficient except a single *Kambata*. The 12.0% G6PDd prevalence among the *Anuak* was also substantial compared to the almost nil ‘highlanders’. The differences were statistically significant in both cases (*p* < 0.0001). The odds of being G6PD-deficient were 4.9 [95% CI 0.635-38.00] and 3.9 [95% CI 0.481-31.418] times higher for the *Nuer* and *Anuak* ethnic groups compared to the ‘highlanders’, respectively (*p* < 0.0001) (Table 2). When the G6PDd prevalence for the *Nuer* and *Anuak* was compared from within the difference was also significant.

**Table 1:** **Prevalences of G6PDd among outpatients of Gambella hospital, southwest Ethiopia by malaria status, sex and ethnic group, 2013**

G6PD	Sex		Malaria		Ethnic group							
		N	Positive no (%)	Negative no (%)	*Anuak*no (%)	*Nuer*no (%)	*Oromo*no (%)	*Amhara*no (%)	*Hadya*no (%)	*Tigre*no (%)	*Kambata*no (%)	*Dawuro*no (%)
Normal	Male	192	117 (60.9)	75 (39.0)	38 (19.7)	59 (30.7)	22 (11.4)	21 (10.9)	14 (7.3)	16 (8.3)	11 (5.7)	11 (5.7)
	Female	224	119 (53.1)	105 (46.8)	42 (18.7)	67 (29.9)	24 (10.7)	20 (8.9)	27 (12.0)	10 (4.4)	18 (8.0)	16 (7.1)
Deficient	Male	18	17 (94.4)	1 (5.5)	7 (14.5)	10 (55.5)	00 (0.0)	00 (0.0)	00 (0.0)	00 (0.0)	1 (5.5)	00 (0.0)
	Female	15	13 (86.7)	2 (13.3)	4 (26.6)	11 (73.3)	00 (0.0)	00 (0.0)	00 (0.0)	00 (0.0)	00 (0.0)	00 (0.0)
Total		449	266 (59.2)	183 (40.8)	91 (20.2)	147 (32.7)	46 (10.2)	41 (9.1)	41 (9.1)	26 (5.8)	30 (6.6)	27 (6.0)
*p*-value	0.354		0.000		0.000

**Table 2:** **Logistic regression analysis of sex and ethnic group for G6PDd among malaria suspect outpatients in Gambella hospital, southwest Ethiopia, 2013**

	Binary logistic regression		Multiple logistic regression	
	UOR (95% CI)	*p*-value	AOR (95% CI)	*p*-value
**Gender**				
Female	1		1	
Male	1.4000 (0.678-2.853)	0.354	1.459 (0.669-2.944)	0.371
**Ethnic group**				
*Nuer*	4.9 (0.635-38.00)	0.000	4.6 (0.589-35.79)	0.000
*Anuak*	3.9 (0.481-31.418)	0.063	3.7 (0.454-30.08)	0.063
*Amhara*	0.00	0.059	0.00	0.059
*Oromo*	0.00	0.044	0.00	0.044
*Tigre*	0.00	0.139	0.00	0.139
*Dawuro*	0.00	0.131	0.00	0.131
*Hadya*	0.00	0.059	0.00	0.059
*Kambata*	1		1	

G6PDd: glucose-6-phosphate dehydrogenase; AOR: adjusted odds ratio, UOR: unadjusted odds ratio, CI: confidence interval.

While G6PD-deficient males who were malaria smear positive were 94.4% (17/18), females of the same category were 87.6% (13/15). Thirty (90.9%) out of the 33 G6PD-deficient participants were malaria smear positive which was significantly higher than the incidence of malaria among normal individuals for the enzyme (*p* < 0.0001). However, the mean parasite density of G6DP-deficient individuals (1.64 (±1.55) was significantly lower than that of G6DP-normal individuals (2.97 (±1.22) (*p* < 0.0001). Similarly, the mean Hb concentration in G6DP-deficient individuals (14.15 (±3.50) was significantly higher than that of normal ones (12.44 (±2.70) (*p* = 0.006). Further, the mean HCT of G6PD-deficient individuals (37.86 (±8.609) was significantly higher than that of normal participants (43.13 (±10.54) (*p* = 0.005). But the mean values of MCV, MCH and MCHC were not significantly different between the two groups (*p* > 0.05) (Table 3).

**Table 3:** **Mean ± SD parasitaemia, Hb, HCT and RBC indices in relation to G6PD status among malaria suspected outpatients in Gambella hospital, southwest Ethiopia, 2013**

G6PD Status	Parasite density/log10 μl	Hb (g/dl)	HCT (%)	MCV (fl)	MCH (pg)	MCHC (%)
Normal	2.97 ± 1.22	12.44 ± 2.70	37.86 ± 8.609	85.51 ± 6.68	26.87 ± 5.58	31.98 ± 2.50
Deficient	1.64 ± 1.55	14.15 ± 3.50	43.13 ± 10.54	85.75 ± 5.84	28.12 ± 4.92	32.43 ± 1.43
p-value	0.000	0.006	0.005	0.834	0.201	0.149

G6PD: glucose-6-phosphate dehydrogenase; Hb: haemoglobin concentration; HCT: haematocrit; MCV: mean corpuscular volume; MCH: mean corpuscular haemoglobin; MCHC: mean corpuscular haemoglobin concentration; RBC: red blood cell; SD: standard deviation.

Seven (21.2%), 1 (3.0%), 9 (27.2%) and 7 (21.2%) individuals were macrocytic, microcytic, hypochromic and microcytic-hypochromic, respectively, among the G6PD-deficient (Table 4). From the non-deficient; 31 (7.4%), 4 (0.96%) 78 (18.7%) and 29 (6.7%) were macrocytic, microcytic, hypochromic and microcytic-hypochromic respectively. The differences between the two groups for the respective parameters were significant.

**Table 4:** **Prevalence of various anaemia types and G6PD status among malaria suspect outpatients in Gambella hospital, southwest Ethiopia, 2013/2014**

Anaemia type G6PD status	Microcytic no (%)	Macrocytic no (%)	Hypochromic no (%)	Microcytic-hypochromic no (%)
Normal	31 (7.4)	4 (0.96)	78 (18.7)	29 (6.7)
Deficient	7 (21.2)	1 (3.0)	9 (27.2)	7 (21.2)
*p-*value	0.000	0.316	0.000	0.000

## Discussion

In this study, *P. falciparum, P. vivax* and mixed infections accounted for 96.6*,* 2.6 and 0.8% of malaria cases, respectively, indicating the overwhelming occurrence of *P. falciparum* in the study population. This result is comparable to a previous report in the area [21] which was 88.9% for *P. falciparum* and 11.1% for *P. vivax* cases. However, the overall malaria prevalence reported by these authors was much lower (13.5%) than that of the current study (59.2%). On the other hand, results from elsewhere in Ethiopia attribute only 60–70% of the cases to *P. falciparum* and the rest to *P. vivax* [22, 23]. The high malaria prevalence in this study shows that the disease is a year-round problem in Gambella despite the undergoing control activities and the fact that the survey was conducted outside the peak transmission season. This may suggest that either the control strategies in use are ineffective and/or some specific local predisposing factors to malaria are impacting. Malaria prevalence did not significantly correlate with participant age or sex but ethnic group. The *Nuer* and *Anuak* were more frequently infected compared to the ‘highlanders’ combined. Environmental and differences in exposure to infectious mosquito bites, among other things, may account for this apparent difference.

The prevalence of G6PDd was 7.3% in this study. This figure is relatively lower compared to the world health organization (WHO) report for SSA which is as high as 35% [12]. The prevalence of G6PDd significantly varies among different countries and regions or even localities for several reasons [24, 25]. For instance, in southern European countries, such as Greece [26] and Italy [27], G6PDd prevalence ranges from 1–30 and 0–3% respectively. A study in India reported G6PDd prevalence of 27.5% in males and 12.8% in females [28]. G6PDd prevalence in Yemen was 7.1% [29] and in the neighbouring Saudi Arabia it was 9.05% [30]. It is widely believed that isolated populations have higher G6PDd cases [26, 31].

Although the frequency of G6PDd was higher in males (4.0%) than in females (3.3%) in the present study, the difference was not statistically significant. Since the gene of the enzyme is on the X-chromosome and males are hemizygous, a single allele of the gene would suffice to express the deficient phenotype. In light of this, a higher frequency of the deficient phenotype is expected in males than in females. Since females inherit two copies of the X-chromosome and therefore have two populations of red blood cells (RBCs), each expressing one of the two G6PD alleles they carry (homozygous females), two deficient alleles are required to render them G6PD-deficient phenotypically. From this point of view, the present study seems to be contrary to what is theoretically expected. However, diagnosing heterozygote females that express two different RBC populations (normal and deficient) to the enzymatic activity level is highly challenging, as the deficiency can be masked by cells expressing normal activity. G6PDd is relatively reliably detected in males [32, 33] and diagnostics for heterozygous females are altogether more complex and uncertain. That is perhaps why G6PDd in females is underreported in many epidemiological studies. Indeed there are studies that reported a narrow variation of G6PDd prevalence between boys and girls with a *p*-value just slightly below the 0.05 level [34].

The prevalence of G6PDd significantly varied between ethnic groups in Gambella area; the highest frequency being among the indigenous *Nuer* and *Anuak* ethnic groups. This must be taken into account in planning primaquine/tafenoquine use to eliminate malaria especially in the rural areas of Gambella where the natives exclusively reside. A similar study in Nigeria recorded some variation in G6PDd with respect to ethnic affiliation though the effect was not significant possibly due to the relatively small number of *non-Yoruba* children in the study, as the authors suggested [35]. In a study from India the G6PDd prevalence ranged from 0 to 27% and a clear variation was noted between different castes, tribes and ethnic groups [36].

The significantly higher occurrence of malaria smear positivity among G6PD-deficient individuals, compared to the non-deficient, observed in this study suggests lack of correlation between G6PDd and protection from clinical malaria. But the significantly lower mean parasite density, higher Hb and HCT values among the deficient shows the protective role of G6PDd against severe malaria. In fact, the literature is not concordant regarding the role of G6PDd in malaria. G6PDd was associated with protection against severe malaria in males but not in heterozygous females [37]. Contrarily, others reported the protection of heterozygous females but not the hemi/homozygous with a more severe degree of G6PDd [38]. Still there is evidence for protection from symptomatic malaria for both hemi/homozygous and heterozygous individuals [39]. G6PDd is weakly associated with asymptomatic malaria infection [40, 41]. High malaria parasitaemia and mortality was observed among G6PD-deficient patients in Iran [42]. In another recent study the odds of malaria infection was not significantly lower in children with the deficient phenotype [34]. *In vitro* studies are equally conflicting [43, 44]. Since G6PD expression is vital for survival of *Plasmodium* inside the host cell the parasite may synthesize its own G6PD within deficient RBCs [45]. This may partially explain why G6PDd is not associated with protection from malaria at least in some settings including the present one. The method of G6PDd assessment can possibly affect the association studies of malaria susceptibility due to G6PDd [46].

It is well-known that G6PDd contributes to macrocytic, microcytic and hypochromic anaemia. In microchromic anaemia the RBC are usually also hypochromic. Further, rapid RBC turnover from G6PDd haemolysis can trigger mild macrocytosis. This may explain the significantly higher proportion of G6PD-deficent individuals for these different types of anaemia compared to the non-deficient in the present study. However, the results should be interpreted with caution as there are several other causes of macrocytic and other types of anaemia. Multiple factors like nutrition, infection, medication, haemoglobinopathies and overall health status may influence G6PD activity [47]. Since the MCV and MCH values for microcytic (MCV <80 fL)*,* macrocytic (MCV >96 fL) and hypochromic (MCH <27 pg) anaemia are comparable especially with that of thalassaemia [48], it may be misleading to conclude that the asymptomatic G6PDd is responsible for the observation in this study. Although little specific information is available concerning haemoglobinopathies in general, Gambella is part of the global thalassaemia map [49]. Thus, future studies are warranted to clearly understand the role of asymptomatic G6PDd on haematological parameters and malaria in relation to haemoglobinopathies in the study area. Though rapid qualitative tests such as the CareStart™ G6PDd screening tests seem more feasible for mass screening in remote endemic areas genotyping G6PD and various haemoglobinopathies is also necessary to explore the local variants. Overall, comprehensive understanding of the relationship between primaquine/tafenoquine dose and risk of G6PDd-related haemolysis is required to allow deploying or withholding these drugs in future malaria control or eradication plans in the study area and beyond.

## Conclusions

G6PDd prevalence as determined by measuring the phenotype (enzyme activity), is considerable among the indigenous Nilotic population. Despite control efforts being made, malaria remains highly prevalent among outpatients in Gambella hospital questioning the efficacy of control strategies in use. There was no significant association between G6PDd and protection from malaria incidence *per se*. The G6PDd report presented here can contribute to the evidence-base for weighing risk and benefit in formulating primaquine/tafenoquine treatment strategies that could greatly accelerate the elimination of malaria transmission in the study area. Population-based genotyping could be important to identify the distribution of dominant variants in the region.

### Limitations of the study

The study did not use quantitative or semi-quantitative screening methods such as spectrophotometric ultraviolet (UV). As a result, the WHO’s five classes (levels) of G6PD activity could not be determined among the study participants. Besides, because of the fact that a molecular method was not used, the underlying prevalent variants and specific mutations in the G6PD-deficient individuals could not be addressed.

## Abbreviations

ANOVA: Analysis of variance
EDTA: Ethylene diamine tetra acetic acid
G6PDd: Glucose-6-phosphate dehydrogenase deficiency
Hb: Haemoglobin
HCT: Haematocrit, km: kilometer
MCV: Mean corpuscular volume
MCH: Mean corpuscular haemoglobin
MCHC: Mean corpuscular haemoglobin concentration
μl: Micro liter
PCV: Packed cell volume
SNNPR: Southern Nations, Nationalities and Peoples’ Region
SSA: Sub-Saharan Africa
SPSS: Statistical package for social sciences
UK: United Kingdom
USA: United States of America
UV: Ultraviolet
WBC: White blood cell
WHO: World Health Organization.
